# Early and Late Shift of Brain Laterality in STG, HG, and Cerebellum with Normal Aging during a Short-Term Memory Task

**DOI:** 10.1155/2013/892072

**Published:** 2013-02-27

**Authors:** Hanani Abdul Manan, Ahmad Nazlim Yusoff, Elizabeth A. Franz, Siti Zamratol-Mai Sarah Mukari

**Affiliations:** ^1^Diagnostic Imaging and Radiotherapy Program, School of Diagnostic Science and Applied Health, Faculty of Health Sciences, Universiti Kebangsaan Malaysia, Jalan Raja Muda Abdul Aziz, 50300 Kuala Lumpur, Malaysia; ^2^Department of Psychology and fMRIotago, University of Otago, William James Building, 275 Leith Walk, Dunedin 9016, New Zealand; ^3^Audiology Program, School of Rehabilitation Sciences, Faculty of Health Sciences, Universiti Kebangsaan Malaysia, Jalan Raja Muda Abdul Aziz, 50300 Kuala Lumpur, Malaysia

## Abstract

Evidence suggests that cognitive performance deteriorates in noisy backgrounds and the problems are more pronounced in older people due to brain deficits and changes. The present study used functional MRI (fMRI) to investigate the neural correlates of this phenomenon during short-term memory using a forward repeat task performed in quiet (STMQ) and in noise: 5-dB SNR (STMN) on four groups of participants of different ages. The performance of short-term memory tasks was measured behaviourally. No significant difference was found across age groups in STMQ. However, older adults (50–65 year olds) performed relatively poorly on the STMN. fMRI results on the laterality index indicate changes in hemispheric laterality in the superior temporal gyrus (STG), Heschl's gyrus (HG), and cerebellum, and a leftward asymmetry in younger participants which changes to a more rightward asymmetry in older participants. The results also indicate that the onset of the laterality shift varies from one brain region to another. STG and HG show a late shift while the cerebellum shows an earlier shift. The results also reveal that noise influences this shifting. Finally, the results support the hypothesis that functional networks that underlie STG, HG, and cerebellum undergo reorganization to compensate for the neural deficit/cognitive decline.

## 1. Introduction

Studies of memory and aging suggest that some functions are impaired in the elderly, whereas other functions are altered only slightly or not at all [1]. Normal age-associated memory decline is not uniform and some cognitive changes are likely to begin in early adulthood. The previous literature on cross-sectional and longitudinal studies suggests that subtle memory changes can begin as early as the early or middle twenties and continue to decline linearly with age [2, 3]. Short-term memory (STM), for example, appears to remain relatively stable until about the age of 70, at which point it begins to drop [3, 4]. Furthermore, normal aging is associated with decline of cognitive performance [1, 5], and these age-related alterations are linked to changes in brain structure and function. One example of the alteration in brain structure and function is a compensatory right side activation in older adults for tasks that are normally left-side lateralized in young adults. This is thought to be related with age-related cognitive decline which affects the right hemisphere more than the left hemisphere. The effect is proposed to be due to grey/white matter ratio which is greater in the left compared to the right hemisphere [6, 7]. Furthermore, research on white matter and grey matter loss during healthy aging also reported a 3%–5% linear reduction per decade in brain areas [8]. Using the voxel-based morphometry (VBM) approach, several magnetic resonance imaging (MRI) studies have reported a consistent pattern of age-related grey matter volumetric reductions in the human neocortex, involving temporal association cortices; these include the superior temporal gyrus (STG) and Heschl's gyrus (HG) [9–11]. In addition, there have been relatively consistent reports of age-related grey matter changes in the cerebellum, basal ganglia, and thalamus [12–15]. Good et al. [13] found a linear decline in global grey matter volume with age and several focal areas of relatively greater age-associated loss. These regions included the cerebellum, middle frontal gyrus, and HG. The reduction in grey matter and white matter volume will contribute to decline in cognitive processing. Age-related declines are thought to undergo a series of deleterious changes which are thought to lead to deficits in cognitive processing including both increases and decreases in brain activity compared to that in the younger, less-impaired brain [16]. Thus, it is important to evaluate these brain areas during cognitive tasks given their sensitivity to aging [17]. Additionally, healthy aging causes chemical and physiological changes which are suggested to contribute to the reorganization of the brain function [16, 18]. This reorganization of the brain may also be explained with the decline-compensation hypothesis [19]. This hypothesis states that the aging brain will underrecruit task-relevant brain regions and recruit additional cortical resources compared to younger brain. Underrecruitment should be a dominant outcome if aging reduces the hemodynamic response or alters its coupling to neural activity. Moreover, additional recruitment of brain areas in older adults suggests that the proper cognitive strategy is used to modify the effects of aging [20].

In cortical processing of auditory stimuli, various age-related changes are found. The most common problem in aged people is related to the difficulty in discriminating speech sounds in noisy environments [21, 22]. This is particularly problematic given that most speech interactions in everyday conditions occur in noisy backgrounds [17, 23, 24]. Moreover, in the noisy environment the demand of attention resources may increase. This is presumably due to mechanisms involved in suppression of irrelevant information and concentration on the task [25, 26]. Older adults are more distracted by novel irrelevant sounds than younger adults, indicating deterioration to the cognitive and auditory processes in relation to aging. In order to assess age-related decline in cognitive performance, a forward repeat span task (FRT) was used in the present study. This task was adapted from that described by Light and Anderson [27], designed to investigate short-term memory (STM) capacity [28, 29]. FRT was used due to its involvement in cognitive processes that require the involvement of STM and attention processes [30]. The use of a verbal form of FRT enables an investigation on both auditory and STM processing [31].

The present study tests two hypotheses by examining STM task in quiet and in 5 dB SNR on four groups of participants with age range between 20 and 65 years. If a mature human brain is capable of retaining a great deal of plasticity, it raises the possibility that cortical reorganization may occur during normal aging, and we might expect to see compensation or overrecruitment of contralateral brain areas which are not activated or less activated in the younger group. Secondly, if the presence of 5 dB SNR background noise severely affects brain reorganization processes compared to that in the quiet background, we might expect to see compensation or overrecruitment of contralateral areas occurring earlier compared to that in quiet.

## 2. Materials and Methods

### 2.1. Participants

Fifty-one Malay male right-handed adults (as indicated by the Edinburgh inventory) [32] with age range of 20 to 29 years (mean 27 years, SD 2.18), 30 to 39 years (mean 33 years, SD 2.18), 40 to 49 years (mean 45 years, SD 2.28), and 50 to 65 (mean 59 years, SD 2.49) (as in Table 1) participated in this study. All participants were native Malay speakers and reported no history of psychiatric or neurological disorders and no current use of any psychoactive medications. Each participant's health status was examined through an interview prior to the experiment. From self-report assessment, no participant had auditory problems. The older adults (group 50 and above) were also given the mini-mental status examination (MMSE) [33] and all of them scored in the normal range between 28 and 30. After full explanation of the nature and risks of the study, informed consent was obtained according to the protocol approved by the Institutional Ethics Committee (IEC) of Universiti Kebangsaan Malaysia. (reference no.: UKM 1.5.3.5/244/NN-075-2009).

### 2.2. Data Acquisition

Functional MRI scans were conducted in the Department of Radiology, UKM Medical Centre, using a 1.5 tesla magnetic resonance imaging (MRI) system (Siemens Avanto) equipped with functional imaging options and echo planar imaging capabilities. A radiofrequency (RF) head coil was used for signal transmission and reception. Prior to each functional imaging scan, an MRI structural scan was obtained. T1-weighted multiplanar reconstruction (MPR) spin-echo pulse sequence was collected with the following parameters: TR = 1240 ms, FOV = 250 mm ×250 mm, flip angle = 90°, matrix size = 128 × 128, and slice thickness = 1 mm. 

Functional images were then acquired using a gradient echo-echo planar imaging (GRE-EPI) pulse sequence. Each whole brain acquisition consisted of 21 axial slices, which comprised all brain regions including the cerebellum. The following parameters were used: repetition time (TR) = 2000 ms, echo time (TE) = 50 ms, field of view (FOV) = 192 × 192 mm, flip angle (*α*) = 90°, matrix size = 128 × 128, and slice thickness = 5 mm with 1.25 mm gap. A sparse imaging paradigm was used to avoid the interference of scanner sound with the stimulus [34]. The same procedures and protocols of the data acquisition have been given elsewhere [35]. 

### 2.3. Stimuli and Materials

Stimuli consisted of series of natural speech words produced by a Malay male adult voice and were digitally recorded (Sony digital voice editor), stored, and edited (Adobe Audition 2.0). The multitalker babble noise stimulus was originally recorded from five volunteers reading different passages simultaneously and was edited so that the intensity level was 50 dB. The intensity level of the short-term memory task in quiet (STMQ) was 55 dB. For the short-term memory task in babble noise (STMN), the signal-to-noise ratio (SNR) was 5 dB throughout the presentation. The same stimuli have been used elsewhere [35]. 

### 2.4. Paradigm and Procedure

**Figure 1:**
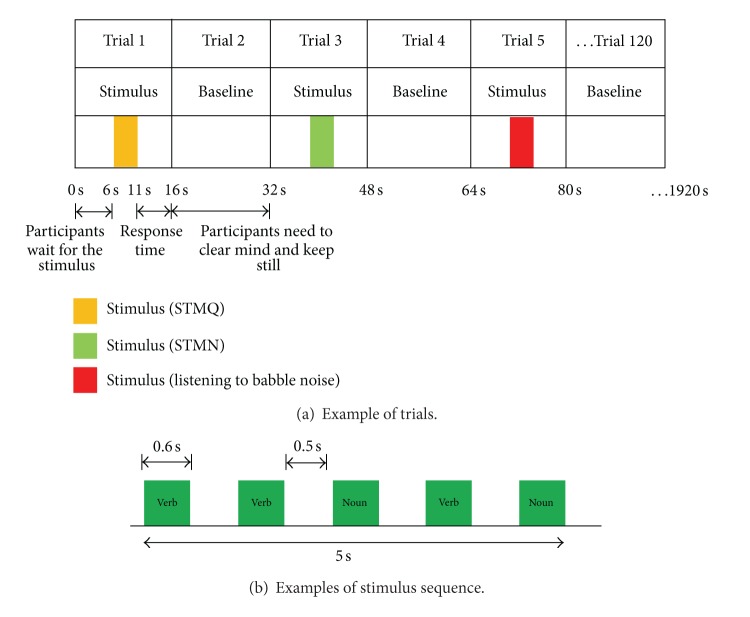
(a) Stimuli were presented in four different conditions: STMQ; STMN; babble noise; baseline (quiet). The sequence of the conditions was fixed; STMQ-baseline-STMN-baseline-Babble Noise-baseline. Total duration of each trial is 16 s. During stimulus trials, stimuli were presented at the 6th second and lasted approximately 5 s, and participants were given 5 s to repeat forward all the words presented. (b) Illustration of stimulus train consisting of a sequence of five unrelated familiar words (verbs and nouns were randomly selected) to produce STMQ and STMN conditions.

The paradigm and procedure of the present study were similar to that in our previous published study [35]. As in Figure 1(a), there were 120 trials in total with 16 s duration for each trial. There were four experimental conditions: (i) 20 trials listening to babble noise (N), (ii) 20 trials performing STM task in 5 dB SNR (STMN), (iii) 20 trials performing STM task in quiet (STMQ), and (iv) 60 rest with no stimuli (Q). The sequence of conditions used during the study was fixed, N-Q-STMN-Q-STMQ-Q-N, for the primary reason that reaction time is faster when using fixed sequences [36]. Total scan time was 32 minutes.

Figure 1(b) shows the experimental trial for STMQ and STMN conditions. A total of 40 (2-syllable and 3-syllable) verbs and nouns which were unrelated familiar Malay words were randomized to produce 40-trial sets. Five consecutive stimuli each with 0.6 s duration separated by 0.5 s silent gap made up a 5 s stimulus train. During a trial, the stimuli were presented at the 6th second and lasted approximately 5 s. For STMQ and STMN conditions, participants were instructed to repeat forward all the words presented. 

### 2.5. fMRI and Behavioural Procedures

Before a participant entered the fMRI scanner, instructions about the task were explained in detail, and instructions were to focus with an otherwise clear mind throughout the procedure and to keep still. During scanning, participants lay comfortably in a supine position in the MR scanner. An adjusted head holder restricted head movement. Auditory stimuli were presented through earphones. During scanning, for STMQ and STMN conditions, participants were given 5 s to repeat forward aloud all the words. Each individual participant's scores were recorded manually by an experimenter in the console room (i.e., number of correct forward repetition trials).

### 2.6. Data Analysis

Behavioural results on the forward repeat task (FRT) with background noise (5 dB SNR) and without noise were analysed in terms of performance accuracy. Paired *t*-tests were used to analyse the effects of noise on the FRT performance for all participants. ANOVA with Tukey's post hoc analysis was used to assess differences in behavioural performance on four groups of participants. Finally, linear regression analysis was used to assess the pattern of behavioural performance in association with aging in the different groups.

To derive activation maps corresponding to the different tasks, our sparse-imaging data were analysed in a manner similar to procedures in our previous published study [35]. fMRI data were analysed using MATLAB 7.4—R2008a (Mathworks Inc., MA, USA) and Statistical Parametric Mapping (SPM8) (Functional Imaging Laboratory, Wellcome Department of Imaging Neuroscience, Institute of Neurology, University College of London, UK; http://www.fil.ion.ucl.ac.uk/spm/). The first two images of every EPI-recording session were discarded to account for the approach to steady state in the MR signal. Prior to image analysis, each participant's raw data of fMRI scan were motion-corrected and normalized, similar to the previous study [37]. The amount of absolute motion did not exceed 1 mm for any participant. Data were further analysed using a 12 parameter nonlinear normalization into the MNI-reference state as implemented in SPM8, and with smoothing (FWHM = 6 mm). The fMRI data were analysed according to the general linear model as implemented in SPM8. With regard to the different conditions, three regressors were included in the design: (i) STMQ, (ii) STMN, and (iii) N. The regressors were convolved using the hemodynamic response function as provided in SPM8. Statistical analysis was performed using a mixed effects model; fixed effects analysis was used for single participant analysis and random effects analysis for group analysis. For group analysis, contrast images were computed for each participant, and then one-sample *t*-tests were performed. A cortical brain region is regarded as significantly activated only if a minimum cluster size of 10 voxels was reached at a corrected value *P*_FWEcorr_ < 0.001. Voxels or clusters with *t* values higher than 3.5 were included in the region-of-interest (ROI) analysis using WFU PickAtlas [12]. In each participant, the activated voxels in each of the ROIs were collected and used to derive the activated volume for each condition for further analysis. Activated volumes in the anatomically defined ROI were compared between tasks (STM in quiet and in 5 dB SNR) using paired *t*-tests and between groups using ANOVA. To test whether activation in regions with significant age-related differences is functionally relevant to the tasks in all groups of participants, a correlation analysis between activated volume and the accuracy in behavioural performance was performed. 

The laterality index (LI) was calculated using the formula LI = (*V*_*L*_ − *V*_*R*_)/(*V*_*L*_ + *V*_*R*_), in which *V*_*L*_ is the number of the activated voxels in the left hemisphere, and *V*_*R*_ is the number of activated voxels in the right hemisphere. The LI ranges from −1 to 1, with −1 to 0 indicating right hemisphere dominance and 0 to 1 indicating left hemisphere dominance [38].

## 3. Results

### 3.1. Behavioural Scores

ANOVA analysis (SPSS 20.0 statistical software package) was used to investigate significant main effects (20–29 year old, group 1; 30–39 year old, group 2; 40–49 year old, group 3; and 50–65 year old, group 4) on the forward repeat task (FRT) (in quiet and in 5 dB SNR). Analyses on noise and in 5 dB SNR were conducted separately and are shown in Table 1. The effects indicated that neither conditions revealed a significant [effect of group] (*P* < 0.05). 

A paired *t*-test was conducted to examine if the mean FRT in quiet and in noise in all groups of participants would differ. The result revealed that the effect of noise on FRT was only significant in participants in group 4 (*P* = 0.001, *t* = 4.533); other groups showed no significant differences (all *P* < 0.05). These results suggest that through the adjustment of the test parameter, task difficulty was reasonably matched for the four age groups. Therefore any differences between groups on the imaging data are more likely due to the effects of aging than to differences in task difficulty.

### 3.2. fMRI Data

The primary goal of the present study was to explore brain activity and the tendency for a laterality shift in four groups of participants performing a short-term memory task in noise (STMN) and in quiet (STMQ), in relation to healthy aging.

#### 3.2.1. Activated Volume

The activated volumes of each age group for each task are given in Table 2 (listening to babble noise), Table 3 (STMQ), and Table 4 (STMN). To examine the specific differences in activation between STMQ and STMN, *t*-tests were used to compare the activated volume of a given region of interest (ROI) in each participant (*P* < 0.05). The results show no significant differences in brain activity between STMQ and STMN in any age group. The laterality index was used to calculate the brain laterality in all tasks (listening to babble noise, STMQ, and STMN) in all four groups of participants. Results indicate that there are changes in brain laterality during aging. Brain areas which are normally leftward asymmetries in young adults during the short-term memory task change to rightward asymmetries in older participants. The brain areas showing such effects include the superior temporal gyrus (STG), Heschl's gyrus (HG), and cerebellum. In contrast, other activated areas including the middle temporal gyrus (MTG), precentral gyrus (PCG), and postcentral gyri (post-CG) remain stable during aging. Results also indicate that the laterality shift varies from one region to another; STG, for example, shows late shifting while cerebellum shows an early shift. This may suggest that aging influences certain areas more than others. More details on the activations and laterality shifting are presented in the following section.

#### 3.2.2. Listening to Babble Noise (N)

N condition was used to evaluate the activation pattern on auditory processing during N task in normal aging brain. The same areas of STG and MTG were used in STMQ and STMN. Table 2 tabulates the number of activated voxels (NOV), coordinates of maximum intensity, and the *t* values of a second-level random effects analysis on four groups of participants. Significant activation in bilateral STG (group 1: left *t* = 6.06, right *t* = 5.09; group 2: left *t* = 6.79, right *t* = 6.52; group 3: left *t* = 8.42, right *t* = 10.33; group 4: left *t* = 7.00, right *t* = 9.27) and MTG (group 1: left *t* = 6.6, right *t* = 5.22; group 2: left *t* = 5.92, right *t* = 4.69; group 3: left *t* = 7.56, right *t* = 8.91; group 4: left *t* = 6.1, right *t* = 6.24) was observed.

Results of the laterality index show that there is shifting in brain laterality during aging. This shifting shows left hemisphere dominance in the younger group and right hemisphere dominance in the older group. Both STG and MTG show that the laterality shifting starts in group 3, suggesting its role in compensating for deficits in cognitive processing during aging.

#### 3.2.3. STMQ and STMN

Comparison between STMQ and STMN in all groups of participants reveals that there is no significant difference in brain activity in all groups of participants (Tables 3 and 4); group 1 (*P* = 0.194, *t* = 1.382), group 2 (*P* = 0.112, *t* = −1.728), group 3 (*P* = 0.939, *t* = 0.078), and group 4 (*P* = 0.349, *t* = 0.978). 

Results on the laterality index demonstrate that there is reorganization of brain laterality in both STMQ and STMN, leftward asymmetries in young group which change to rightward asymmetries in the older group. Areas involved in this change are the STG, cerebellum, HG, and PCG. The result also reveals that these laterality shifts vary from one region to another. In STMQ, results indicate that STG (group 1: left *t* = 6.61, right *t* = 7.23; group 2: left *t* = 13.13, right *t* = 12.45, group 3: left *t* = 12.72, right *t* = 14.3; group 4: left *t* = 6.04, right *t* = 8.11) starts to become more rightward asymmetries in group 4. However, for STG in STMN (group 1: left *t* = 5.78, right *t* = 5.73; group 2: left *t* = 12.98, right *t* = 11.39; group 3: left *t* = 18.25, right *t* = 12.69; group 4: left *t* = 8.47, right *t* = 7.46), the laterality shift starts earlier (group 3). For the cerebellum, during STMQ (group 1: left *t* = 5.34, right *t* = 6.4; group 2: left *t* = 8.18, right *t* = 9.69; group 3: left *t* = 10.23, right *t* = 8.86; group 4: left *t* = 7.00, right *t* = 4.59) and STMN (group 1: left *t* = 5.04, right *t* = 5.61; group 2: left *t* = 10.14, right *t* = 11.63; group 3: left *t* = 6.41, right *t* = 7.39: group 4: left *t* = 7.00, right *t* = 5.89), the changes in hemisphere laterality start as early as in group 2 participants. For PCG, during STMQ (group 1: left *t* = 5.09, right *t* = 4.64; group 2: left *t* = 8.71, right *t* = 9.24; group 3: left *t* = 8.79, right *t* = 6.66; group 4: left *t* = 7.83, right *t* = 5.68), the results show no changes in brain laterality. However, in STMN (group 1: left *t* = 4.47, right *t* = 5.24; group 2: left *t* = 10.06, right *t* = 9.83; group 3: left *t* = 7.38, right *t* = 6.66; group 4: left *t* = 5.01, right *t* = 4.84), the changes begin to show up in group 4. For HG, changes of brain laterality both in STMQ (group 1: left *t* = 5.58, right *t* = 0; group 2: left *t* = 6.08, right *t* = 6.92; group 3: left *t* = 6.55, right *t* = 5.57; group 4: left *t* = 4.61, right *t* = 6.69) and STMN (group 1; left *t* = 5.72, right *t* = 0, group 2: left *t* = 5.86, right *t* = 6.73; group 3: left *t* = 8.96, right *t* = 6.16; group 4: left *t* = 0, right *t* = 5.44) also are shown in group 4.

## 4. Discussion

### 4.1. Behavioural Scores

The demographic and performance accuracy obtained from four groups of participants on the forward repeat task (FRT) (in quiet and in 5 dB SNR) is shown in Table 1. Results indicate that there is no significant difference across groups on performance accuracy on any task. We suggest that aging has minimal effects on both FRT (in quiet and in 5 dB SNR conditions) tasks. Both tasks comprise passive phonological loop and short-term memory (STM) processing in which participants need to memorise a series of words in a short period of time and repeat them forward. Our behavioural result is consistent with Dobbs and Rule [4], indicating that STM is relatively stable compared to other types of memory processing, and remaining relatively stable until about the age of 70, at which point it begins to drop [2]. Furthermore, our behavioural study is supported by the results which indicate that the human brain has a tremendous capacity to repair itself; no matter how old the individual is, the brain can modify its structure and function to compensate for the age-related cognitive decline [4]. 

Paired *t*-tests were conducted to examine if the mean FRT in quiet and in noise differed in the four groups of participants. Results revealed that the effect of noise on FRT was only significant for group 4. Participants in other groups show no significant differences between performance in quiet and in background noise. This may suggest that the deficit of performance in the presence of background noise in group 4 might be related to older adults having difficulties in paying attention to relevant information and ignoring irrelevant information in the environment due to aging [39, 40]. The present result is congruent with the fact that aging is associated with difficulty in discriminating speech sounds in noisy environments due to attention deficits [21, 41]. We further suggest that this problem also might be linked with degradation of cognitive processing due to aging. Therefore, we propose that the impairment in performance in group 4 participants in the noise condition might be linked with deficits in attention in which they are less able to discriminate between background noise and the target stimulus.

### 4.2. Behavioural Scores in Relation to fMRI

**Figure 2:**
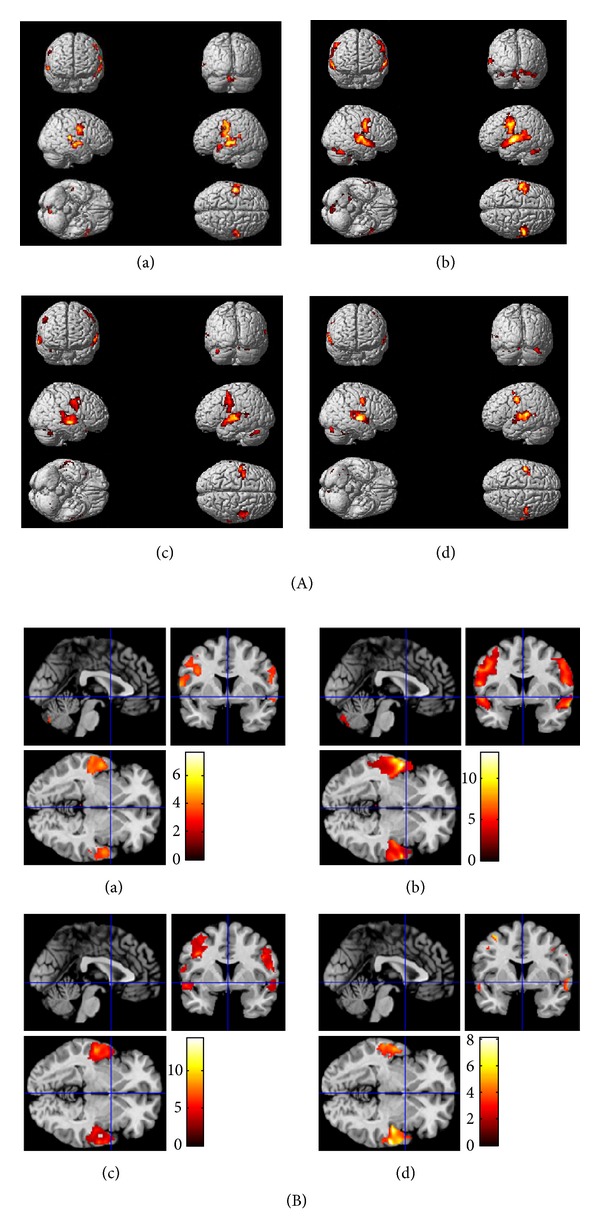
(A) Statistical parametric maps (SPMs) obtained from four groups of participants on random effects analysis (*P*_FWEcorr_ < 0.001) on word-based STMQ. Whole brain maps show activation in (a) 20–29 year olds, (b) 30–39 year olds, (c) 40–49 year olds, and (d) 50–65 year olds (note: left side of the brain is on the left: neurological conventions). (B) Brain activation from the four groups of participants on random effects analysis (*P*_FWEcorr_ < 0.001) for word-based STMQ, shown for (a) 20–29 year olds, (b) 30–39 year olds, (c) 40–49 year olds, and (d) 50–65 year olds with findings overlaid onto structural brain images, displayed in transverse, sagittal, and coronal planes (note: left side of the brain is on the left: neurological conventions).

**Figure 3:**
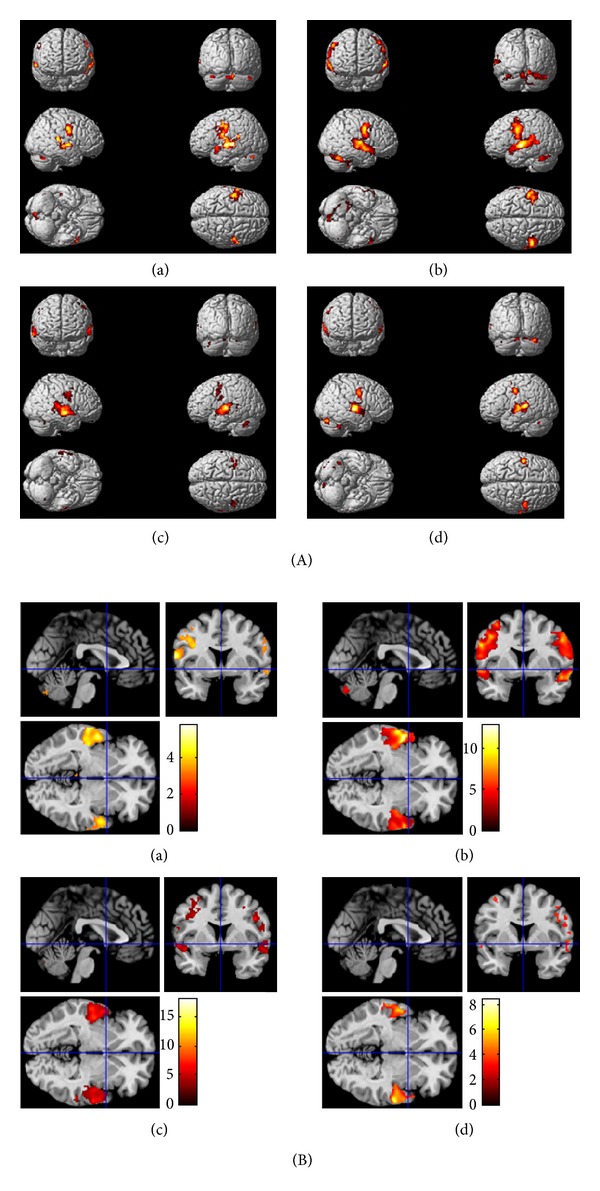
(A) Statistical parametric maps (SPMs) obtained from the four groups of participants on random effects analysis (*P*_FWEcorr_ < 0.001) for word-based STMN. Whole brain maps show activation in (a) 20–29 year olds, (b) 30–39 year olds, (c) 40–49 year olds, and (d) 50–65 year olds (note: left side of the brain is on the left: neurological conventions). (B) Brain activation from the four groups of participants on random effects analysis (*P*_FWEcorr_ < 0.001) for word-based STMN. Effects are shown for (a) 20–29 year olds, (b) 30–39 year olds, (c) 40–49 year olds, and (d) 50–65 year olds overlaid onto structural brain images, displayed in transverse, sagittal, and coronal slices (note: left side of the brain is on the left: neurological conventions).

fMRI results reveal that brain behaves differently with different age groups (Tables 2 and 3). Results from the laterality index calculation revealed changes in spatial distribution of brain activation patterns, with a leftward asymmetry in the younger groups (Figures 2(A) and 2(B)), which appears to shift to more rightward asymmetry in the older groups as in Figures 3(A) and 3(B). These laterality changes can be seen in the STG, HG, and cerebellum. However, the behavioural performances (as in Table 1) show no significant differences across the four groups of participants. In the present study, we propose two possibilities to account for changes in hemispheric laterality without changes in task performance. First, we suggest that the aging brain uses different brain circuitry in order to accomplish the same tasks and recruits contralateral brain areas in order to compensate for neural degradation. As people age, they continue to generate new nerve cells and also use more parts of the brain than do young adults [40]. Thus, the reorganization of the brain throughout an individual's life essentially compensates somewhat for neural deficits. Other possibility is that cognitive functions in younger adults are more intact compared to those of older adults. Thus, the demand is high in older adults in responding to the same cognitive task or stimulus. Hence, overrecruitment appears in the right hemisphere of the brain due to the high processing demand and in response to altered function in other brain regions. These could be linked with neural areas in the right hemisphere declining faster than in the left hemisphere, consistent with the differences in grey and white matter ratios in the two hemispheres [7]. 

## 5. fMRI

### 5.1. Listening to Babble Noise (N)

The N condition imposed the fewest processing demands, as the participants only needed to listen to the babble noise presented binaurally. Activation on STG and MTG was expected given the use of nonverbal auditory stimuli, and results are similar to those observed in normal hearing listeners on tasks using nonverbal stimuli which also demonstrate involvement of the STG [42]. Results indicate that all groups of participants activate the same areas of the brain (Table 2). However, results on the laterality index calculation show that there are differences in the shifts of brain laterality with leftward asymmetries in group 1 and group 2, and more rightward asymmetries in group 3 and group 4 for both the STG and MTG. These age-related differences in the pattern of neural activity suggest that the brain reorganises functional processing by recruiting additional contralateral areas in the brain, something akin to a compensation mechanism. This is supported by the fact that the same general brain regions are used to complete the task [43]. Furthermore, there is evidence that when one network of neurons dies, the brain can sprout new connections, creating another network [44]. Thus, the increase of neural activity in the right hemisphere in groups 3 and 4 is consistent with the notion of a compensation strategy to ameliorate effects of neural decline.

### 5.2. STMQ and STMN Conditions

As in Tables 3 and 4, activation of the STG and HG in both STMQ and STMN was expected given that auditory stimuli were used and these areas have been associated with auditory processing [45, 46]. As we asked our participants to remember the words presented, and at the same time hold the information in memory for subsequent reporting, PCG and post-CG were also activated. These areas are often reported in verbal STM and play important roles in rehearsal of verbal information [47]. Moreover, the need to attend might also account for activation of the cerebellum. Traditionally, the cerebellum has been thought to be responsible for motor coordination and balance but recently this area is hypothesized to be involved in attention during cognitive tasks [48, 49].

The brain is a dynamic and adaptable system; thus, it might modify its structure and networks in order to compensate for cognitive decline. Notably, in comparing effects across the four groups of different ages STMQ and STMN, results reveal that there is no specific pattern of a gradual increase or decrease of neural activity in particular regions with age. Rather, results are consistent with a reorganization of neural patterns characterized by a shift in hemispheric laterality in the STG, cerebellum, HG, and PCG. Results also reveal that these laterality shifts vary from one region to another. Finally, noise also influences the laterality changes.

In STMQ, results indicate that the STG began to shift from leftward to rightward laterality at around the age of participants in group 4 (50–65 years). However, for the STG in STMN, the laterality shift started earlier in age (group 3: 40–49 years). We suggest that the earlier shift in laterality of STG in the 5 dB SNR could be related to increasing demands of cognitive processing in the aging brain. Furthermore, with aging, individuals experience a decreased ability in discriminating speech, even when they have normal hearing acuity [50]. This difficulty is more obvious when the speech is presented in the presence of competing signals (background noise). We propose that this problem might be related to an inability to discriminate between noise and target stimuli, which is a normal aging phenomenon [51]. In HG, the laterality shift in both conditions began to show in group 4 participants. We suggest that, during the task, the demand on this area is equally utilized in both conditions and that noisy backgrounds do not influence this processing area. This implies that age-related cognitive decline affects HG in both hemispheres equally, at least in the present task.

The cerebellum in both conditions, STMQ and STMN, begins to show a shift in hemisphere laterality in people as young as 30–39 years (group 2 participants). It is reasonable to suggest that this early shift is related to the cerebellum being more sensitive to aging effects compared to other activated brain areas. This also might suggest that attention plays a major role in the present task, which is to hold the information and suppress irrelevant information and concentrate on the task particularly in the present of background noise. Older adults are more distracted by novel irrelevant sounds than younger adults, indicating a relatively larger temporary capture on their attentional resources. Deficits in attention and declines in the cognitive system have been shown to be related to anatomical atrophy [6] and it is also likely that attentional abilities are affected during aging. This is in line with a previous study reporting that older adults with declining sensory perception also have compromised attention [52]. It has also been suggested that the ability to comprehend a cognitive task in challenging situations (in noisy backgrounds) is influenced by both auditory and cognitive capacities [53]. 

Age-related changes of brain laterality in older groups are suggested to be a cognitive strategy to ameliorate some of the effects of aging [20]. On the other hand, this adaptive strategy is to maintain performance in the face of decline in the function of canonical neural circuits and may therefore represent evidence of compensation [43]. A related possibility is that additional brain areas in the contralateral hemisphere are recruited because older adults encounter more difficulty and expend more effort on tasks compared to younger adults. In our view, the present findings provide rather convincing support for the idea that changes in brain laterality can be compensatory in the elderly [44]. We further suggest that older brains do things differently, even when behavioural performance is comparable to that of younger adults [54].

Collectively, age-related changes in brain structure and function may contribute to poor sensory and cognitive function [6, 54]. Previous studies found age-related alterations in the anterior-posterior scalp distribution, suggesting a possible decrease in efficiency of attentional processes or that aging alters auditory processing associated with attentional regulation [55, 56]. We suggest that aging-related cognitive deficits can be explained by the deficit in attentional abilities. It has been shown that noisy backgrounds capture the attention of the aged more easily than that of the young. Finally, these results imply that the aging brain might initiate changes in neural mechanisms which lead to a global reorganization of task-specific neurocognitive networks in order to compensate for decline due to the aging process. We also propose that age-related changes in brain regions including the STG, HG, and cerebellum are task dependent and are influenced by effects of aging. 

## 6. Conclusion

The present study examined and compared age-related neural correlates in four age groups of participants on an auditory STM task performed in quiet and in (5 dB SNR) multitalker babble noise, in the entire brain using fMRI. The present study highlights two prominent findings. Although all groups of participants activated the same neural areas in processing the STM task, the groups differed in the way such processing took place. Such processes reveal differential multiple patterns of brain activity across groups, with a shift from a leftward laterality to a more rightward laterality with aging. Changes in hemispheric laterality varied across the different brain regions, with the STG and HG showing late shifting, and the cerebellum showing earlier shifting, with respect to the age of the participants comprising each group. This strongly suggests that the laterality shifts in brain regions vary depending on the sensitivity of the regions to demands of the present tasks. The present study also reveals that noise influences the laterality shift. Furthermore, results support the hypothesis that the functional networks that underlie attention and auditory processing undergo reorganization during aging and these processes are task dependent. These findings provide novel insights into the patterns of neural compensation in the aging brain. 

## Figures and Tables

**Table 1:** Demographic and performance data obtained from 51 participants.

Group number: age range	(1) 20–29	(2) 30–39	(3) 40–49	(4) 50–65
*N*	15	15	10	11
Actual age (range)	23–29	30–37	41–47	50–65
Age (mean ± SD)	27 ± 2.18	33 ± 2.18	45 ± 2.28	59 ± 2.645
Years of education (mean ± SD)	14.8 ± 0.79	15.4 ± 1.50	13.9± 3.16	13 ± 2.49
Word-based STMQ, accuracy rate (mean ± SD)	16.49 ± 2.28	17.73 ± 2.26	14.44 ± 4.5	14.64 ± 3.57
Word-based STMN, accuracy rate (mean ± SD)	17.2 ± 2.92	18.13 ± 2.21	14.44 ± 7.07	11.73 ± 4.28

STMQ: short-term memory task in quiet, STMN: short-term memory task in noise.

**Table 2:** Anatomical area, brain hemisphere, *t* value, coordinates of maximum intensity (*x*,  *y*,  *z*), and number of activated voxels obtained from group analysis (*P*_FWEcorr_ < 0.001), comparing four groups of participants in listening to babble noise condition (N).

Anatomical area	Hemisphere	*t* value	Coordinate (*x*, *y*, *z* mm)	NOV	*t* value	Coordinate (*x*, *y*, *z* mm)	NOV	*t* value	Coordinate (*x*, *y*, *z* mm)	NOV	*t* value	Coordinate (*x*, *y*, *z* mm)	NOV
			20–29			30–39			40–49			50–65	

	L	6.06	−66, −26, 6	1131	6.79	−56, −2, −2	1165	8.42	−64, −18, 6	1079	7	−58, −26, 8	963
		5.99	−56, −16, 2		5.48	−60, −22, 2	39	8.02	−52, −6, −8		6.3	−58, −12, 10	
STG		5.09	46, −20, 2	1120	6.52	44, 8, 20	824	10.33	54, 8, −12	2058	9.27	62, −14, −2	1307
	R	5.06	56, −8, 0		5.54	44, −10, −10		9.75	66, −14, 0		9.05	50, −10, −6	
		4.97	62, 2, −2		5.4	60, −4, −4		9.18	54, −4, −6		6.62	56, −2, −12	

	L	6.6	−66, −38, 8	525	5.92	−62, −14, 0	246	7.56	−58, −6, −8	700	6.1	−58, −32, 8	343
		6.16	−66, −28, 6		4.66	−60, −4, −8	73	7.41	−64, −22, 2		5.35	−52, −20, 0	
MTG		5.22	70, −34, −2	326	4.69	66, −26, −2	62	8.91	60, −8, −16	1161	6.24	58, 0, −14	463
	R	5.06	66, −52, 8		3.5	66, −18, −8		8.07	58, −46, 4		5.27	68, −32, 0	
		4.95	66, −46, −8					7.2	62, −54, 8		4.99	64, −22, −6	

NOV: number of activated voxels, STG: superior temporal gyrus, MTG: middle temporal gyrus, L: left, R: right.

**Table 3:** Anatomical area, brain hemisphere, *t* value, coordinates of maximum intensity (*x*,  *y*,  *z*), and number of activated voxels obtained from group analysis (*P*_FWEcorr_ < 0.001), comparing four group of participants in word-based STMQ.

Anatomical area	Hemisphere	*t* value	Coordinate (*x*, *y*, *z* mm)	NOV	*t* value	Coordinate (*x*, *y*, *z* mm)	NOV	*t* value	Coordinate (*x*, *y*, *z* mm)	NOV	*t* value	Coordinate (*x*, *y*, *z* mm)	NOV
			20–29			30 −39			40 −49			50 −65	

		6.61	−60, −12, 12	138	13.13	−56, −6, 0	1504	12.72	−58, −20, 2	571	6.04	−58, −32, 10	253
	L	5.09	−36, −36, 10	49	9.81	−62, −14, 2		8.63	−56, −8, 6		5.86	−50, −30, 6	
STG		4.79	−50, 12, −18	89				6.87	−56, −4, −4		5.35	−62, −22, 4	
		7.23	46, −24, −4	25	12.45	64, −10, 6	1192	14.3	54, −16, −8	368	8.11	64, −18, 0	422
	R	6.56	56, −30, 12	110	9.9	62, −4, −2		6.44	60, −28, 6	42	4.83	60, −32, 4	13
		4.63	58, −8, −2	35				5.82	66, 32, 14	20			

	L	5.59	−54, −28, −4	122	9.9	−62, −14, 0	246	8.49	−58, −20, 0	135	5.91	−58, −32, 8	38
MTG		4.95	−60, −10, −6		8.48	−58, −10, −6	53	5.14	−46, −24, 0	10	5.87	−64, −28, −2	68
	R	4.92	48, −22, −8	25	6.15	69, −20, −4	23	11.42	52, −16, −10	108			
								10.75	66, −18, −10				

		5.09	−50, −4, 46	68	8.71	−54, −6, 34	737	8.79	−42, 2, 38	138	7.83	−42, −8, 44	84
	L	4.64	−40, 2, 32	18	8.52	−46, −10, 30		5.86	−52, −6, 50	28	6.02	−36, −2, 58	21
PCG		4.35	−54, −8, 30	10				5.85	−36, 2, 54	37			
		4.64	50, −8, 36	23	9.24	50, −4, 40	393	6.66	46, 0, 34	120	5.68	44, −6, 42	51
	R				8.57	46, −8, 34		5.95	48, 8, 42	10			
								5.91	56, 0, 22	18			

	L	5.34	−4, −74, −24	25	8.18	−24, −62, −28	286	10.23	−40, −68, −28	51	—	—	—
Cerebellum		4.68	−4, −38, −6	17	5.62	−4, −74, −16	183	5.61	−42, −58, −34				
	R	6.4	26, −64, −30	35	9.69	34, −60, −30	628	8.86	36, −58, −32	108	4.59	38, −78, −24	19
		—	—	—	5.4	24, −40, −50	18	6.44	26, −68, −26		—	—	—

		7.67	−62, −10, 14	300	9.56	−56, −6, 16	530	8.19	−58, −2, 20	39	6.96	−44, −10, 40	99
	L	5.29	−44, −12, 38		8.47	−54, −6, 36		4.94	−40, −12, 38	27	5.94	−46, −8, −48	
Post CG		4.67	−54, −10, 28		8.18	−46, −10, 32							
	R	5.25	56, 10, 22	145	8.19	56, −4, 30	227	6.31	56, −2, 22	73	—	—	—
		5	50, −10, 34										

	L	5.58	−32, −30, 10	37	6.08	−36, −30, 14	185	6.55	−48, −16, 6	42	4.61	−42, −26, 10	11
Heschl gyrus		4.76	−58, −12, 8	13	5.72	−56, −10, 8							
	R	—	—	—	6.92	40, −20, 6	124	5.57	60, −4, 6	10	6.69	64, −4, 6	12
					6.31	60, −8, 6					5.69	46, −16, 4	19

“—”: not significant.

STMQ: short-term memory task in quiet, NOV: number of activated voxels, STG: superior temporal gyrus, MTG: middle temporal gyrus, PCG: precentral gyrus, Post-CG: postcentral gyrus, L: left, R: right.

**Table 4:** Anatomical area, brain hemisphere, *t* value, coordinates of maximum intensity (*x*,  *y*,  *z*), and number of activated voxels obtained from group analysis (*P*_FWEcorr_ < 0.001), comparing four group of participants in word-based STMN.

Anatomical area	Hemisphere	*t* value	Coordinate (*x*, *y*, *z* mm)	NOV	*t* value	Coordinate (*x*, *y*, *z* mm)	NOV	*t* value	Coordinate (*x*, *y*, *z* mm)	NOV	*t* value	Coordinate (*x*, *y*, *z* mm)	NOV
			20–29			30–39			40–49			50–65	

		5.78	−50, 12, −18	54	12.98	−56, −6, 0	1431	18.25	−58, −18, 4	624	8.47	−56, −10, 4	270
	L	5.77	−42, −40, 14	52	11.26	−62, −14, 2		10.2	−48, −16, 2		6.93	−48, −30, 4	
STG		5.14	−54, −12, −2	136	10.19	−48, −38, 14		8.21	−64, −26, 10		5.89	−62, −24, 4	
		4.51	−38, −30, 4	51									
	R	5.73	44, −26, −4	10	11.39	66, −10, 6	1262	12.69	52, −12, −6	730	7.46	62, −18, 0	354
		5.47	53, −32, 10	83	11.23	62, −2, −4		8.76	46, −18, −10		5.58	68, −16, 10	

	L	5.81	−54, −28, −4	179	10.73	−62, −14, 0	269	8.12	−66, −20, −10	104	6.96	−58, −10, −6	27
MTG		4.93	−48, −22, −6		5.13	−54, −50, 6	35	7.83	−58, −18, 0		6.03	−50, −34, 8	74
	R				6.93	68, −20, −4	30	12.31	66, −18, −10	141	—	—	—
								10.31	46, −20, −10				

PCG	L	4.74	−48, −8, 42	50	10.06	−56, 0, 34	807	7.38	−28, −4, 48	28	5.01	−44, −8, 44	35
		4.63	−38, 2, 30	17	8.58	−48, 6, 48							
	R	5.24	50, −8, 38	58	9.83	52, −2, 40	491	6.66	44, 0, 34	15	4.84	48, −6, 40	44
					8.66	46, −8, 34							
					6.4	52, −2, 28							

	L	5.04	−28, −60, −32	46	10.14	−24, −60, −26	610	6.41	−38, −66, −30	45	—	—	—
		4.63	−22, −64, −26										
Cerebellum		5.61	24, −66, −28	24	11.63	36, −60, −30	899	7.39	24, −66, −24	48	5.89	38, −78, −24	63
	R	5.15	8, −80, −28	22	7.7	22, −38, −48	15	5.4	30, −56, −34	14			
					5.04	30, −36, −38	13						

	L	5.35	−62, −2, 18	77	8.42	−50, −8, 32	466	8.15	−58, −4, 20	41	5.06	−46, −10, 40	58
Post CG		5.31	−44, −12, 38	114	8.3	−56, −6, 16		6.22	−56, −8, 44	16	4.64	−48, −8, 48	
	R	5.5	50, 10, 36	131	9.75	42, −10, 32	271	—	—	—	4.24	56, −6, 36	12
		4.35	54, −4, 30		7.89	52, −4, 30							

		5.72	−34, −30, 6	14	5.86	−40, −20, 2	166	8.96	−48, −16, 6	58	—	—	—
	L				5.47	−56, −20, 2		5.61	−38, −22, 10	18			
Heschl gyrus					5.32	−36, −30, 14							
		—	—	—	6.73	42, −20, 4	142	6.16	50, −16, 4	48	5.44	62, −2, 6	10
	R				6.24	46, −24, 12					5.27	46, −16, 4	39
					6.08	50, −14, 4							

“—”: not significant.

STMN: short-term memory task in noise, NOV: number of activated voxels, STG: superior temporal gyrus, MTG: middle temporal gyrus, PCG: precentral gyrus, Post-CG: postcentral gyrus, L: left, R: right.
